# Study on the Applicability and Modification of the Design Hourly Volume on Rural Expressways Considering Holiday Traffic Polarization

**DOI:** 10.3390/ijerph19169897

**Published:** 2022-08-11

**Authors:** Fangchen Ma, Jinliang Xu, Chao Gao, Yufeng Bi

**Affiliations:** 1 College of Highway, Chang’an University, Xi’an 710064, China; 2 Shandong Provincial Communication Planning and Design Institute Group Co. Ltd., SHANDONG HI-SPEED Group, Jinan 250000, China

**Keywords:** DHV, holiday traffic polarization, 30 HV, expressway design

## Abstract

The design hourly volume (DHV) of traffic based on the 30th highest hourly volume (30 HV) of the year has been widely applied in expressway design in various countries to balance the benefit and economy of expressway engineering. However, this design method has barely changed since it was first adopted in China, which may be contrary to the rapidly changing traffic macroenvironment. In this study, annual hourly traffic volume (HV) data pertaining to expressways in East China, Southwest China and Northwest China were collected. Based on the descending order of the obtained HV and HV factor data, the distribution patterns of the traffic demand throughout the year and peak hours were analyzed. The distribution characteristics of the HV, typicality of 30 HV and applicability of the DHV factor were investigated. It was found that severe polarization occurred in the HV distribution in China. The actual 30 HV factor is more than 0.5 times the recommended value in the specification. Continued use of the current DHV would result in more than 200 h of inefficient travel time, 5.7 times more than expected, with the DHV factor is currently no longer applicable in China. Furthermore, the annual 30 HV value loses its typical status. Depending on the level of local economic development, using 10 HV factor or 80 HV factor as the new DHV factor can better alleviate the congestion problem. This study determines the reasons for the widespread congestion issues in China from the perspective of expressway design, which is beneficial to adjust the basis of expressway design in China.

## 1. Introduction

The design hourly volume (DHV) of traffic, as the basis of road design, is widely considered in expressway design systems in many countries. The DHV directly affects the construction scale, construction cost, traffic efficiency, and traffic safety of road facilities. An inappropriate DHV can lead to traffic congestion, causing serious gas and noise pollution, while increasing the likelihood of traffic accidents, and endangering public health.

The original DHV derivation was proposed by Peabody in 1941. Peabody found that when the annual 8760 h are arranged according to the hourly traffic volume (HV) in descending order, the HV distribution changes from sparse to dense. Based on this finding, he proposed that as a design basis, a DHV value higher than the 30th highest hourly volume (30 HV) is not economical, whereas a value lower than the 50th highest hourly volume (50 HV) will result in limited cost savings at the notable expense of capacity. Subsequently, the industry has gradually established a method for the estimation of 30 HV based on historical data [1]. By the end of the last century, the predictability and consistency of the relationship between the annual average daily traffic volume (AADT) and 30 HV was recognized by expressway engineers worldwide [2]. The ratio of these two indicators (referred to as the design HV factor in certain regions), as a key parameter to calculate the DHV based on AADT predictions, has been applied in many countries, such as the United States, China, and Japan [3,4,5]. The specific value range is determined by each country according to domestic historical data. China considers the product of the predicted AADT, directional split factor, and DHV factor (i.e., K) as the one-way designed HV to guide expressway design.

The product of AADT, direction inhomogeneity coefficient (D) and DHV factor (K) is used as DHV to guide expressway design in China, whereby the value range of parameter D is 0.5–0.6, taking into account the difference in traffic volume in both directions. According to regional traffic characteristics, China is divided into six regions, and the recommended K values for rural and suburban areas are given respectively.

The purpose of adopting the 30 HV as the DHV is to control the road congestion time by approximately 30th hour, but this purpose is often not achieved in reality. Especially in China, expressway congestion lasts longer than 30 h [6,7,8]. In previous studies, the reasons for the deviation in DHV are roughly divided into two categories. One reason is that the ratio of 30 HV to AADT is not sufficiently stable. The 30 HV-to-AADT ratio fluctuates to a certain extent due to seasonal factors and traffic activities. The other reason is that the AADT estimation method is not accurate enough, resulting in estimated values that are higher or lower than the actual values [9,10]. However, the above studies cannot explain the systematic DHV underestimation under the current traffic conditions in China.

China first implemented the DHV to guide expressway design in 1997. However, the macroscale traffic background and public travel characteristics have changed in China. In particular, the Chinese government has increased the number of holidays by 11 days since 1999, and the policy of free expressways for private cars on holidays was implemented in 2012 [11]. This series of policies has resulted in high-intensity public travel conditions during holidays, resulting in congestion [12,13,14,15]. However, it should be noted that despite the notable change in traffic background, the recommended value range of the DHV factor K in China (8~13.5%) has remained almost unchanged over the initial K value range (9.5~13.5%) [16]. This range may be unreasonable, and further improvements are urgently needed.

Among the attempts made by scholars to improve the accuracy and applicability of the DHV against the background of specific traffic demand changes such as holidays, the most concerning aspect is the AADT prediction accuracy. Scholars have mostly improved the AADT prediction method in various aspects, such as traffic investigation mode, prediction model, and algorithm optimization, according to holiday-specific measured data and traffic characteristics [17,18,19,20,21,22,23]. In addition, based on observation data, Sharma and Walters found that the proposed direction inhomogeneity coefficient D could significantly impact the DHV calculation process [24,25,26]. Although the deviation in DHV caused by holiday traffic conditions has been recognized by many scholars, most scholars still adopt the recommended 30 HV value conforming to the rule verified in the original derivation, while the empirical relationship between 30 HV and AADT has not changed greatly. Moreover, the current DHV factor is still adopted. This may be contrary to the current conditions in China. Bailey and Sharma studied the limitations of the applicability of the 30th HV as the DHV, but they mainly focused on the volatility of the ratio of the 30 HV to the AADT [10,27], rather than the change in travel characteristics.

This paper mainly focused on the following four issues:

- Are the relevant DHV indicators still applicable?
- Against the current HV distribution background, does the DHV application environment persist?
- How does holiday traffic affect the hourly traffic distribution and DHV estimation methods? Does the 30 HV indicator typically balance the economy and efficiency under holiday traffic conditions?
- How should the DHV estimation method be modified to enhance the indicator applicability?

To explore the above issues, this paper investigates the annual traffic data of several expressways in northwest, southwest and east China. The inconsistency between the current actual DHV and HV distributions in China was analyzed. Finally, the deviation in the applicability of the DHV method and the underlying causes were examined, and corresponding correction directions were proposed. The findings of this paper elucidate the causes of the current widespread congestion situation in China in terms of design, and this study provides a theoretical basis to resolve this problem. Furthermore, the gas and noise pollution caused by traffic congestion, as well as traffic accidents and public health risks will be reduced. 

## 2. Theoretical Analysis Methods

### 2.1. Data Acquisition and Selection

Annual HV data were obtained from traffic observation stations belonging to the Expressway Management Company in Zhejiang Province, Yunnan Province and Shaanxi Province. These traffic observation stations are equipped with traffic observation equipment satisfying industry requirements and can continuously record 8760 h of road traffic data throughout the year. The data from these traffic observation stations were examined to determine the principle of sample selection. Sample selection adhered to the following principles to obtain representative sample data:

i. The AADT value based on the selected samples should be consistent with the predicted AADT value of road design in general, to exhibit a higher comparative research value. Moreover, traffic congestion caused by traffic overflows should be avoided, to clearly observe the traffic peaks.
ii. Large-scale public events attracting much traffic should not be held in the surrounding areas of the selected expressway during the year of investigation. Geological and climatic disasters or major emergencies affecting the road network traffic distribution do not occur.
iii. Throughput constraints due to upstream or downstream congestion should not exist. Traffic anomalies caused by accidents during the year of investigation do not occur during possible peak traffic periods, and these anomalies thus exhibit a short duration and limited impact.

**Figure 1:**
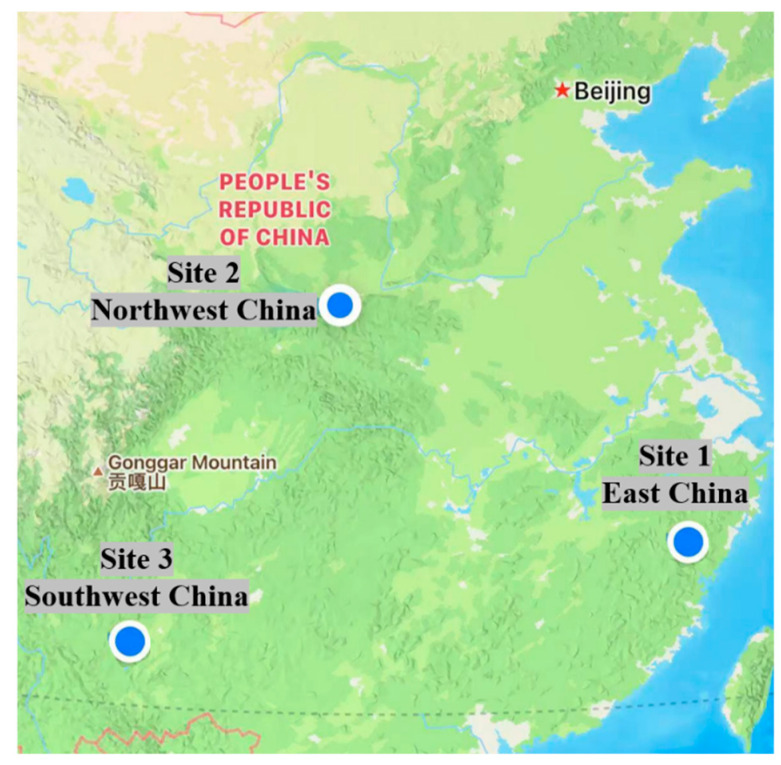
Geographical location of the surveyed expressway.

After investigating the traffic counter data in the last five years in many parts of China, the annual HV data of Liwen Expressway (Site 1), Xixian Expressway (Site 2) and Songkun Expressway (Site 3) from 2017 to 2018 were selected to obtain 52,560 h of traffic data. The three expressways are located in northwest China, southwest China and east China. The geographical locations are shown in Figure 1.

These three expressways are located in typical rural areas; hence, the recommended K values are 13.5%, 13% and 12.5%, respectively [5]. The difference between the AADT value for the selected expressway survey year and the road design-based value was not more than 3%. Although integrity requirements were taken into account in selecting data, a small amount of missing data was still found during data collection. The average traffic volume in the same hour in the previous week and the following week is used as the traffic volume of the missing hour. Although the HV obtained by interpolation is not real survey data, due to the periodicity of traffic fluctuations, there is little difference between the interpolation value and the actual value. The annual total traffic volume after interpolation can basically represent the actual annual total traffic volume.

### 2.2. Analysis Method

China uses unidirectional AADT to calculate DHV. The product of AADT and the direction inhomogeneity coefficient D was used as an estimate of unidirectional AADT. However, some scholars pointed out that the characteristics of the uneven direction of expressways would affect the accuracy of the analysis [24], so this paper directly used the actual unidirectional traffic data instead of the product of AADT and D to make the research more accurate. Therefore, the HV and AADT are unidirectional indicators in the following sections.

In the original derivation of DHV, Peabody sorted the annual HV from large to small, observed the distribution of traffic in peak hours, and determined the DHV [1]. The data acquisition and processing of this method are simple, and the display of traffic volume distribution in peak hours is intuitive and clear. It has become a typical method for analyzing DHV and 30 HV, and is widely used [2,17,18,19]. This study will continue to use this method to explore the impact of current traffic characteristics on hourly traffic volume distribution and DHV selection. In this study, the HV values during the annual 8760 h were sorted in descending order, the descending order HV data of each expressway for two years are averaged, and the annual HV data of three expressways are obtained, to compare the relevant parameters under standard conditions (i.e., the recommended K and 30 HV values) to those based on the sample data to verify the applicability of this indicator. The distribution structure of the peak HV was analyzed to determine whether the general rule of the distribution status is consistent with that in the DHV application environment. The annual HV values based on the sample data were sorted chronologically to study the HV fluctuation characteristics against the holiday traffic background. Combined with the discovered rules and characteristics, DHV correction directions were analyzed, and correction suggestions were proposed.

## 3. Analysis

The study was divided into the following four parts:

- By comparing the DHV and actual data, the applicability of the DHV method was studied against the current traffic background.
- By examining the distribution of the peak HV, the application environment of the DHV method was analyzed.
- According to the fluctuation in the HV against the background of holiday traffic and the peak hour period, the HV distribution characteristics and causes were revealed.
- Based on the above analysis, DHV correction methods were obtained.

### 3.1. Applicability of the DHV against the Holiday Traffic Background

K in the expressway DHV method is the ratio of 30 HV to AADT, and the applicability can be assessed by comparing K to the actual HV/AADT ratio. Table 1 provides the HV/AADT ratio corresponding to 20th hour (i.e., the number of hours associated with the 20th highest hourly volume of the year), 30th hour, 50th hour, the number of hours of the regional recommended value K, the corresponding upper limit ratio (13.5%) and lower limit ratio (8.5%) of K in China.

Table 1 indicates that the actual ratios of 30 HV to AADT in the three regions are 19.6%, 21.6% and 18.6%, respectively, 1.6 times, 1.7 times and 1.4 times of the recommended values K in each region. The average value is 1.5 times that of the upper limit of the DHV factor in China. The ratio of HV to AADT near 30th hour changes notably. In addition, the appropriate traffic volume between 20 and 40 HV can be adopted as the DHV according to the conditions in China [27]. However, the data show that the actual factors of 20 HV, 30 HV and 40 HV are far more than the recommended limit of 13.5% of the DHV factor in China. The corresponding HV/DAADT in the sample ranges from 17.8% to 22.7%, which also greatly differs from the recommended K value.

From the perspective of design, the number of hours corresponding to the recommended K values in the three regions is 201, 237 and 239, respectively. The total traffic volume before the number of hours corresponding to K value accounted for 11.1%, 10.4% and 5.8% of the total traffic volume in the year. The expressways, designed according to the K value of the survey area, are theoretically likely to have more than 200 h of inefficient traffic every year, well above the 30 h expected. At the same time, the traffic volume that leads to abnormal service accounts for 5.8% to 11.1% of the total annual traffic volume, resulting in serious traffic efficiency problems. This result is basically consistent with the current serious expressway congestion issues in China.

The above analysis reveals that the HV distribution has greatly changed. The current HV peak is higher, and the number of peak hours is greater, which preliminarily reflect the occurrence of HV polarization. This situation directly leads to serious K failure in guiding road design. Given the current HV distribution, the DHV method seriously underestimates the current HV peak, leading to poor applicability, which may be the direct reason for the current widespread congestion in China.

### 3.2. Analysis of the Application Environment of the DHV Method

A prerequisite for the DHV method is that the annual HV distribution conforms to the DHV application environment, and the application environment is analyzed in this part.

AADT of Wenli, Songkun and Xixian expressways are 9625 veh, 8746 veh and 11,759 veh, respectively. The first HV were 2768 veh, 2817 veh and 3314 veh, which is 6.9, 7.1 and 6.7 times the annual average HV, respectively. The difference between the peak and average HV values is very large. If the annual average HV value was applied as the DHV, more than 4000 inefficient operation hours per year could occur, thus seriously impairing the traffic efficiency. Moreover, the difference between the peak HV values is large, as the difference between the first HV and 50 HV values is 1000 veh, 1078 veh and 1292 veh, respectively. If the first HV value were adopted as the DHV, the DHV could reach 0.57, 0.62 and 0.64 times the 50 HV value, respectively, an increase in the number of served hours of only 5‰, which is a poor choice in terms of the engineering economy. This is consistent with the rules obtained in the past.

**Figure 2:**
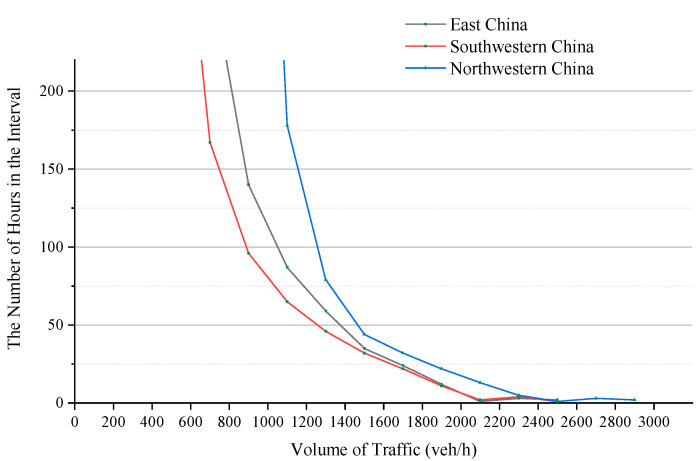
Number of hours in the various traffic volume intervals.

Figure 2 shows the overall distribution characteristics of the HV. The horizontal axis indicates the traffic volume. The vertical axis indicates the number of hours within each interval. Figure 2 further reveals that the number of hours per unit interval with a higher traffic volume is always small, and the total number of hours in the first three 200 veh intervals was 6, 7 and 6, respectively, and the number of hours in the subsequent intervals increased gradually. This means that within these intervals, the increase in DHV by several hundred veh increases the number of hours served by only a few. The overall HV distribution still exhibits a shift from sparse to dense, which conforms to the basic rule of the DHV method.

However, it should be noted that the dispersion in the peak HV notably changes. According to previous studies on the HV peak, the HV distribution dispersion increases with increasing number of hours, and the difference between the first few HV values is often large. With increasing number of hours, the HV distribution becomes increasingly concentrated. In other words, the number of hours in the first 200 veh interval should be very small, and then the number of hours contained in each interval should gradually increase, and the increase speed is faster and faster, and the slope of the curve shows a rapid change. However, it can be seen from the figure that the number of hours contained in the second interval is always higher than that in the third interval, showing opposite changes. Second, each curve starts from the third interval, and the slope of the curve has no obvious change in several intervals. This is not consistent with the traditional rule. We will analyze the reasons below.

Although the distribution of the peak HV is quite different from that in the past, the overall rule of the HV still conforms to the basic rule of the DHV design method. Therefore, it is necessary to choose an appropriate DHV value to avoid long-term large-scale congestion and sharp increases in the project scale attributed to the pursuit of meeting extreme traffic needs. Therefore, although the recommended DHV value is no longer applicable, the application environment and requirements of this method still persist.

### 3.3. Influence of Holiday Traffic on the Peak HV Distribution

The large variation in the peak hour distribution is likely related to the change in the travel demand of people.

**Figure 3:**
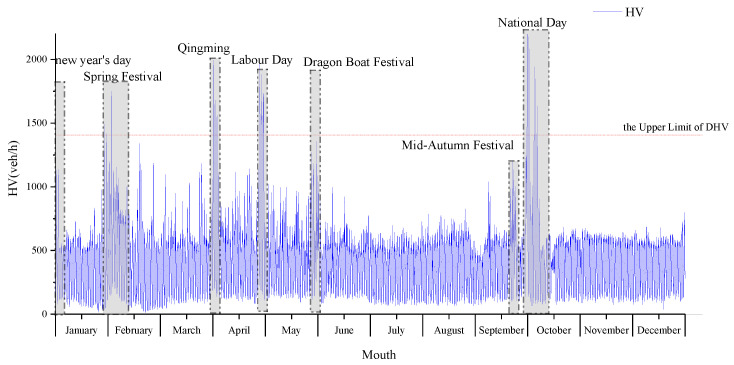
Time series of the hourly traffic volume distribution.

**Figure 4:**
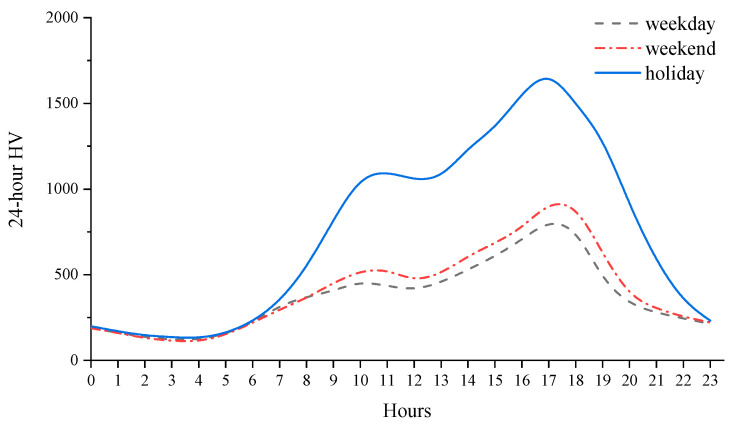
Curves of 24-h traffic volume change on working days, weekends and holidays.

Figure 3 shows a diagram of the annual HV values in chronological order. The horizontal axis indicates the annual 8760 h in chronological order, divided by the month. The vertical axis indicates the HV, and the reference line indicates the DHV of the investigated expressways. China’ s holidays are based on the lunar calendar. Some holidays have different periods in different years, so they should be analyzed in terms of years. Since the data for 2018 and 2017 show very similar patterns, the data for 2018 alone are taken as an example. The figure shows that the occurrence of peak hours can be divided into two situations: one type of peak hour period occurs once within a few days and lasts for one or several hours, and the other type occurs repeatedly during multiple periods within a few days. It should be noted that the peak hours repeatedly occurring during multiple periods were always observed during the statutory holiday period (the start time of the holiday period is actually the end time of the last working day before the holiday period, which includes the evening of the holiday), as marked with framing lines in the graph. According to the obtained statistical results, of the top 30 peak hours, 29 h occurred during the holidays, accounting for 96.7% of the total peak hours. Of the top 100 peak hours, 91 h were observed during the holiday period, accounting for 91% of the total peak hours. The average HV value during the holiday period was 995 veh, which is 2.3 times higher than the average HV value (432 veh) during the nonholiday period. The statutory holiday period generated 91% of the top 100 peak hours in 7.7% of the annual 8760 h. For comparison, data from Songkun Expressway included 87 h during the holiday period, and this value for Luxiaozhai Expressway was 75 h. This reveals that the holiday traffic demand is likely to be an important reason for current peak hours. Figure 4 shows the 24-h traffic curve for all sites over two years on weekdays, weekends, and holidays. It can be seen that during the holidays, the high level of traffic operation time is significantly increased compared with the weekdays and weekends.

Peak hours are often observed during the holiday period. 73.6% of the holiday peak hours occurred in the morning or afternoon of the first day of the holiday period. This demonstrates that the occurrence of holiday peak periods is predictable to a certain extent. In existing studies, the traffic volume is affected by the season, month, period and other factors, resulting in cyclical fluctuations [1,3,4]. Thus, peak hours randomly occur, which may be the main cause of the inverse correlation between the number of peak hours and the dispersion in the peak HV. The above analysis reveals that during the statutory holiday period in China, the travel demand of people surges, and the emergence of peak periods exhibits a certain inevitability. Accordingly, peak hours within the context of holiday traffic can be divided into two categories: one category is attributed to the booming traffic demand during the holiday period, as characterized by a larger number of hours and a concentrated distribution. The other category involves periodic random fluctuations and is characterized by a smaller number of hours and a scattered distribution, which corresponds to previous research results. These two categories constitute the distribution status of the peak HV, which may be the reason for the new distribution characteristics shown in Figure 2.

It should be noted that the top 100 HV values exceeded the DHV value of expressway design, which further illustrates the polarization phenomenon of the HV distribution caused by holiday traffic. The principal cause for the generation of the peak HV has fundamentally changed, which further affects the HV distribution throughout the year and leads to DHV failure. In the following section, this effect is analyzed based on data.

### 3.4. Analysis of the Application Environment of the DHV Method

HV polarization affects the distribution of the peak HV, which exerts an important influence on the DHV method.

**Figure 5:**
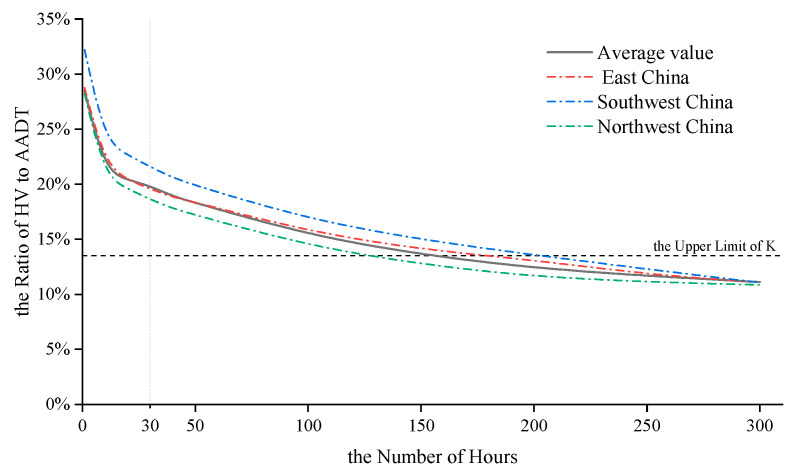
Relationship between the number of peak hours and HV-to-AADT ratio.

Figure 5 shows the HV/AADT ratios in descending order for the top 300 h of the HV. The horizontal axis indicates the number of peak hours, and the vertical axis indicates the HV/AADT ratio. This graph is generally applied as the basis to determine the recommended value K of DHV. In terms of the average data of the three regions, obviously, the number of hours when HV/AADT is higher than the upper limit of recommended value K (13.5%) is far more than 30, which reveals the polarization phenomenon of the HV distribution from another perspective. The figure reveals that the peak HV distribution still transitions from dense to sparse. The first 49 HV values are distributed in the first 10% of the entire range, whereas the second 10% of the entire range contains the next 827 HV values. However, it should be noted that the curve is no longer showing a clear trend from steep to slow in the 30-hour position, as reported in previous research results. Due to the booming traffic demand during the holiday period, this trend appears in position 10. At the same time, the traditional rule that peak HV decline rate decreases rapidly has also changed significantly: HV decreased rapidly in the first 10 h, and the decline rate was very stable in the next dozens of hours.

**Figure 6:**
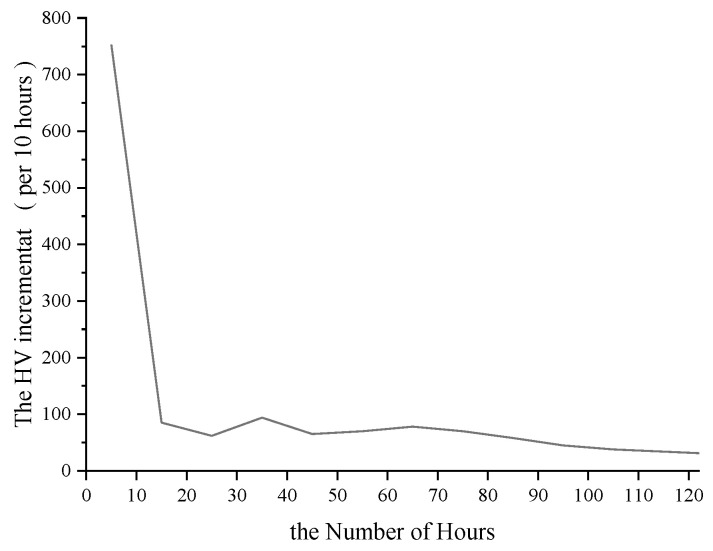
The HV increment of peak hours (per 10 h).

This stable decline rate alters the typicality of the 30th hour position. Figure 6 presents the relationship between the number of hours selected for the DHV and the DHV increment. It can be seen that the difference between the first HV and 10 HV is 752 veh, which is consistent with the traditional rule. However, the HV difference between 20 HV and 60 HV per 10 h is 85 veh, 62 veh, 94 veh, 65 veh, and 70 veh, respectively, and the difference is very small. This reveals that the decline rate of HV is stable in this interval, which is consistent with the distribution characteristics of HV in Figure 2. It is worth noting that the difference between 30 HV and 20 HV is 62 veh, and the difference between 30 HV and 40 HV is 94 veh, where the decline rate even slightly increases. in other words, the traffic volume distribution temporarily exhibits the opposite density trend. This is contrary to the characteristics of the rapid and sharp change in HV decline rate near 30th hour in the traditional rule.

In fact, the characteristic that the HV decline rate did not change significantly until the 70th hour was changed. The difference between 70 HV and 80 HV, 80 HV and 90 HV, 90 HV and 100 HV was 48 veh, 41 veh and 38 veh, respectively. The HV decline rate began to slow down gradually.

Combined with Figure 2 and Figure 4, overall, the decline rate of peak HV still shows a rule from fast to slow. However, the characteristics of the sharp change in HV decline rate near 30th hour in the traditional rule shifted to near 10th hour. After 10th hour, the decline rate of HV is slow and the change is small.

## 4. Discussion

### 4.1. Accuracy of Data Interpolation

The integrity of the annual HV data greatly impacts the analysis results. If part of the hourly traffic was missing, the calculated annual total traffic and average daily traffic could be lower, resulting in a higher HV factor than the actual value. In 8760 h of collected yearly data, due to hardware or software problems and uncontrollable factors, a small amount of missing data is inevitable. The processing steps of missing data generally include random value interpolation, mean or median interpolation and platform interpolation. Compared to the method without interpolation, the methods of random value interpolation and mean or median interpolation could yield AADT values more closely reflecting the actual conditions to a certain extent, but the reliability is difficult to evaluate, and the error between the interpolation and true values exhibits randomness, which could affect HV distribution rule analysis. The platform interpolation method used in this paper is more suitable for HV research. The platform interpolation method uses similar values obtained from the complete data to replace missing values [28], and the HV exhibits fluctuations with monthly, weekly and daily cycles [29]. Therefore, weekly HV data could be considered a complete dataset. With the use of the average traffic volume during the same hour of the previous and following weeks as the traffic volume during the missing hour, the interpolation accuracy could be improved. To verify this method, this paper randomly compared the difference between the traffic volume during a given hour and the average traffic volume during the same hour of the previous and following weeks, and a total of 50 groups were sampled. It was found that the error in this interpolation method remained within 9%, and the influence of every 100 interpolations on the AADT did not exceed 1‰.

### 4.2. Causes of High Peak HV Characteristic Change

Peak HV is no longer strictly distributed from sparse to dense, that is, the decreasing rate gradually decreases from fast to slow. Instead, it drops rapidly in the first 10 h, with little change in rate between the 10th hour and 70th hour. According to past experience with the HV distribution, this characteristic does not generally occur only due to fluctuation randomness [2]. This may well occur because the peak traffic demand includes a new reason: changes in population travel characteristics. The main traffic peak is no longer generated by the past rule of random fluctuations, but the holiday travel peak is caused by the surge in the holiday traffic demand. The peak HV during the holiday period is generally higher than the peak HV due to random fluctuations. Due to the holiday peak, the difference in traffic volume between 10th hour and 70th hour is not significant, and 30th hour may plot in the flat part of the distribution curve, rather than the part with the curve and the transitions from steep to relatively flat. The steady rate of decline between 20HV and 70HV means that there is no significant difference between the number of hours of service that can be gained by increasing DHV and the number of hours of service that can be lost by decreasing DHV. Thus, the 30 HV indicator may have lost its typical status of balancing efficiency and economy.

The prevalence of holiday peaks stabilizes the emergence of traffic peak hours in China, mainly occurring on the first day and evening of the holiday period. The reason may be that people tend to choose to travel at the beginning of the holiday period and return before the end of the holiday period, which may also be the reason why peak hours do not occur on the last day of the holiday period. This feature facilitates the implementation of traffic congestion mitigation measures.

### 4.3. Effect of Polarization of Peak HV and Disappearance of 30 HV Typicality on Selection of DHV

The annual HV value in China may exhibit a serious polarization phenomenon. This phenomenon has altered the previous empirical relationship between 30 HV and AADT, causing the peak traffic conditions based on DHV to be underestimated. To a certain extent, HV polarization is detrimental to the economy of expressway design. Adaptations to the polarization characteristics of the peak hourly traffic to improve the DHV only remain advantageous for short periods, which may be unwise in terms of engineering economy. In contrast, the number of hours corresponding to the recommended value K of DHV is above 200. Ignoring this part of the traffic demand could lead to the public having to endure the current bad traffic congestion conditions. Therefore, raising the DHV is also an urgent consideration.

### 4.4. Suggestions on DHV Correction

The main reason for the normalized congestion of China’s intercity expressway is that the DHV is too small to meet the polarized traffic under the background of holiday traffic. In order to avoid normalized congestion, improving the factor of DHV is the most effective improvement method.

One possible improvement method is to use 10 HV factor and 80 HV factor as the DHV factor of developed and underdeveloped regions, respectively. For example, 10 HV factor is used in East China, namely 0.218 as the DHV factor, and 80 HV factor is used in Northwest China and Southwest China, namely 0.152 and 0.177 as the DHV factors, respectively.

This is due to the characteristics that the decline rate of hourly traffic volume is slowed down near the 10th and 80th hours, so the two locations can balance the traffic efficiency and engineering cost to a certain extent. However, by using 10 HV as DHV, the DHV factor may increase to about 21.3%, which is about 1.7 times higher than the current recommended value, which means that the new rural expressway may increase one or two lanes, and the engineering cost will increase greatly. At the same time, the traffic efficiency and traffic safety will be significantly improved, and the annual inefficient traffic time will be approximately 10 h. This approach may be more applicable in economically developed regions. Moreover, if the DHV is corrected according to the 80 HV, the DHV factor is about 16.2%, which is about 1.3 times higher than the current recommended value. This means that some new rural expressways may add a lane, and the engineering cost increases to a certain extent. At the same time, the traffic efficiency and safety are improved to a certain extent. This method may be more suitable for economically underdeveloped regions.

It should be noted that the DHV factor should generally be determined based on local data. Therefore, when determining the DHV factor of the proposed expressway, the HV factor corresponding to the design hour should be determined after investigating the local annual traffic data. If the data are lacking, the results of this study can be referred to.

## 5. Conclusions

The DHV based on 30 HV greatly impacts the construction scale, construction cost, traffic efficiency and traffic safety of road facilities.

This paper reveals that the surge in the traffic demand likely results in the original DHV-related indicators no longer being applicable under the influence of public holidays and the free expressway policy in China. The main reason for DHV failure is the surge in holiday traffic, which has altered the previous empirical relationship between 30 HV and AADT and the typical status of 30 HV. Compared to previous studies, the results indicate that the peak HV distribution structure exhibits polarization. The peak HV distribution is highly concentrated, and the HV value near 30th hour no longer occurs at its typical position considering the traffic efficiency and construction cost. However, since the overall HV distribution still shifts from sparse to dense, the DHV method remains valuable.

In economically developed regions, the 10th hourly traffic volume can still be used as the design basis. When the economic conditions are limited, the 80th hourly traffic volume can be used. But the DHV factor should be determined in combination with traffic survey.

We observed the failure of DHV in northwest, southwest and eastern China, and put forward some suggestions for improvement, but there are some limitations in the study. First, although we interpolate the missing traffic volume to make AADT more accurate, we are not sure whether these missing values include peak hours. The impact of missing data interpolation remains to be assessed. Second, among the three expressways investigated in this study, Songkun Expressway started to operate for only two years, with its traffic volume still in the growth period, and the traffic volume characteristics are imperfect, which may have a certain impact on the results. In addition, the scientific and reasonable determination of the DHV factor need to clarify such impact at the project scale, and this paper has not yet reached a clear conclusion.

To determine the HV range or the HV based on the DHV method, a large number of detailed investigations should be carried out on the actual HV distribution in various typical regions of China in the future. These investigations should involve more expressways to consolidate and develop the rules found in this research. The range and recommended value of K should be determined according to the background and characteristics of holiday traffic. When revising the DHV method, different design hours should be selected according to various holiday peak characteristics or holiday peak correction factors should be introduced to enhance the DHV applicability in reflecting regional differences in the holiday traffic demand.

## Figures and Tables

**Table 1:** Ratio of HV to AADT (typical number of hours).

Number of Hours	HV/AADT in East China	HV/AADT in Southwest China	HV/AADT in Northwest China	Mean HV/AADT
20	20.4%	22.7%	19.6%	20.4%
30	19.6%	21.6%	18.6%	19.8%
40	18.8%	20.6%	17.8%	18.9%
……	-	-	-	-
126	-	13.5%	-	-
177	13.5%	-	-	-
155	-	-	-	13.5%
201	-	-	13.5% (K)	-
237		12.5% (K)		-
239	12% (K)	-	-	-
456	-	8.5%	-	
516	8.5%	-	-	8.5%
830	-	-	-	-
1029	-	-	8.5%	-

## Data Availability

Restrictions apply to the availability of these data. Data was obtained from Zhejiang Jinliwen Expressway Co. Ltd., Yunnan Kunsong Expressway Development Co. Ltd. and Shaanxi Road Network Management Center. And are available from Fangchen Ma (2019021084@chd.edu.cn) with the permission of Xiaodong Zhang (z17602970421@163.com), Haoru LI (170258941@163.com), and Chenwei Gu (wow693433698@163.com).
